# Poly(methyl methacrylate) with Oleic Acid as an Efficient *Candida albicans* Biofilm Repellent

**DOI:** 10.3390/ma15113750

**Published:** 2022-05-24

**Authors:** Milica Petrović, Marina Randjelović, Marko Igić, Milica Randjelović, Valentina Arsić Arsenijević, Marijana Mionić Ebersold, Suzana Otašević, Irena Milošević

**Affiliations:** 1 Powder Technology Laboratory, Institute of Materials, Ecole Polytechnique Fédérale de Lausanne, 1015 Lausanne, Switzerland; petrovicmilica21@gmail.com; 2 Department of Microbiology and Immunology, Faculty of Medicine, University of Niš, Blvd. Dr Zoran Djindjić 81, 18000 Niš, Serbia; marina.randjelovic@medfak.ni.ac.rs (M.R.); otasevicsuzana@gmail.com (S.O.); 3 Public Health Institute Niš, Blvd. Dr Zoran Djindjić 50, 18000 Niš, Serbia; 4 Department of Prosthodontics, Faculty of Medicine, University of Niš, Blvd. Dr Zoran Djindjić 81, 18000 Niš, Serbia; marko.igic@medfak.ni.ac.rs; 5 Department of Pharmacy, Faculty of Medicine, University of Niš, Blvd. Dr Zoran Djindjić 81, 18000 Niš, Serbia; milica.randjelovic@medfak.ni.ac.rs; 6 National Reference Medical Mycology Laboratory, Institute of Microbiology and Immunology, Faculty of Medicine, University of Belgrade, Dr Subotića 1, 11000 Belgarde, Serbia; mikomedlab@yahoo.com; 7 University of Applied Sciences of Western Switzerland-Hepia, Hes-So Geneva, 1202 Geneva, Switzerland; irena.milosevic@hesge.ch

**Keywords:** oleic acid, PMMA, *C. albicans*, filamentation, biofilm, antimicrobial surface

## Abstract

Poly(methyl methacrylate) (PMMA), widely used in dentistry, is unfortunately a suitable substrate for *Candida* (*C*.) *albicans* colonization and biofilm formation. The key step for biofilm formation is *C*. *albicans* ability to transit from yeast to hypha (filamentation). Since oleic acid (OA), a natural compound, prevents filamentation, we modified PMMA with OA aiming the antifungal PMMA_OA materials. Physico-chemical properties of the novel PMMA_OA composites obtained by incorporation of 3%, 6%, 9%, and 12% OA into PMMA were characterized by Fourier-transform infrared spectroscopy and water contact angle measurement. To test antifungal activity, PMMA_OA composites were incubated with *C*. *albicans* and the metabolic activity of both biofilm and planktonic cells was measured with a XTT test, 0 and 6 days after composites preparation. The effect of OA on *C*. *albicans* morphology was observed after 24 h and 48 h incubation in agar loaded with 0.0125% and 0.4% OA. The results show that increase of OA significantly decreased water contact angle. Metabolic activity of both biofilm and planktonic cells were significantly decreased in the both time points. Therefore, modification of PMMA with OA is a promising strategy to reduce *C*. *albicans* biofilm formation on denture.

## 1. Introduction

*Candida* species cause oral candidiasis, the most common fungal infection in the oral cavity, with a high incidence among diabetic, cancer, oral-prosthetic, and immunosuppressed patients and patients on long-term treatment with antibiotic and corticosteroid therapy [1,2,3]. Especially in the oral cavity of the denture wearer, *Candida* easily colonizes the inner surface of the denture, which is typically made of poly(methyl methacrylate) (PMMA) [4]. This frequently used material is hydrophobic and has a relatively rough surface, facilitating the *Candida* biofilm accumulation [5,6]. However, when *Candida* is in the form of biofilm, it is difficult to treat and remove it since the fungus is encased in an extracellular matrix which protects it from penetration of the antimicrobial agents [7,8,9,10]. Thus, the formed *Candida* biofilm on a denture surface acts as a source of infection, continuously reinfecting oral mucosa, [11,12] leading to the development of *Candida*-associated denture stomatitis (CADS) [13,14,15,16,17,18]. Among *Candida* spp., *Candida albicans* is the most often isolated species that cause infection. However, *C*. *albicans* biofilm formed is difficult to treat with antifungal drugs. In fact, such treatment is usually unsuccessful due to the persistence of the infection, as a consequence of the biofilm formed on the denture surface, but also due to the fact that the resistance of *C*. *albicans* within the biofilm [19].

Hence, novel strategies are required to control biofilm formation by pathogens. An alternative approach can be a modification of the current denture materials with antimicrobial compounds, which could prevent *C*. *albicans* adhesion or biofilm formation on the denture surface such as chlorhexidine, fluconazole, amphotericin B, nystatin, or silver nanoparticles [13,14,17,18,20]. *C*. *albicans* is often resistant to conventional antimicrobial therapy, which compromises using antimicrobial drugs as fillers. The limiting factor in the use of silver nanoparticles is their cytotoxicity, and their possible releasing from the denture due to the pressure during mastication [21,22]. Therefore, as an antimicrobial agents could be considered molecules which are naturally abundant, such as plant-derived ones. Research showed that incorporation of undecylenic acid, natural compound into PMMA provide antifungal properties of modified PMMA. However, undecylenic acid can be cytotoxic for human cells, in concentration which will completely inhibit biofilm formation on the denture [23]. Interestingly, naturally occurring oleic acid (OA) has been recently reported for its antifungal activity [21,22]. 

Importantly, OA prevents *C*.*albicans* transition from yeast to hyphal form [21,22], a crucial step in biofilm formation and invasion of biomaterial. 

To date, there has been no reporting carried out on the usage of OA in treating *Candida* infection especially associated with wearing denture., Furthermore, OA has not been yet used for surface modification of dental materials and there has no reported its cytotoxicity neither antimicrobial resistance. In order to overcome this challenge, and keeping in mind that OA inhibits the transition of *C*. *albicans* yeast to hyphal form and consequently biofilm formation, we proposed to incorporate OA into PMMA in order to obtain a surface that will reduce *C*. *albicans* biofilm formation. In this study, we have incorporated different amounts of OA into the PMMA matrix, with the aim of developing an antibiofilm composite material. The goal was to create material with OA molecule on the surface of modified PMMA, where OA preserves antifungal properties. Since OA is an unsaturated fatty acid, insoluble in water, it is a challenge to incorporate OA in polymers and create a composite in such way that OA still preserves its antifungal properties. Therefore, we studied the physicochemical properties of the composite’s surface, characterized by Fourier- transform infrared spectroscopy (FTIR) and by measuring the water contact angle. Moreover, antimicrobial properties of the PMMA_OA composites were studied with the XTT test by measuring the percentage of metabolically active biofilm (attached to the surface) and planktonic (free-floating) *C*. *albicans* cells. To better understand how OA affects *C*. *albicans*, we studied the effect of OA on the morphology and growth of *C*. *albicans* cells in embedded conditions.

## 2. Materials and Methods

### 2.1. Sample Preparation

PMMA-OA composites were made by physical incorporation of suitable amounts of OA into a mixture of cold polymerized acrylic resin-PMMA and MMA (Triplex Cold, Ivoclar Vivadent, Liechtenstein), in a ratio 13 g PMMA with 10 mL MMA, according to the manufacture instruction [4]. Samples of composites containing 0, 3, 6, 9, and 12 wt% OA were made as solid discs (Ø 20 mm) in Teflon molds for all experiments except for the 2,3-bis(2methoxy-4-nitro-5-sulfophenyl)-5-[(phenylamino)carbonyl]-2H-tetrazolium hydroxide (XTT) test. For the XTT test, composites with 0, 3, 6, 9, and 12wt% OA were made in 24-well tissue culture plates (Falcon 353047). Before the experiment, composites were sterilized under a UV-C lamp for 15 min. 

### 2.2. Physico-Chemicals Characterization of PMMA_OA

#### 2.2.1. Fourier Transform Infrared Spectroscopy (FTIR) 

Chemical characterization of the surface of the PMMA_OA, PMMA, and OA, was carried out by Fourier- Transform Infrared Spectroscopy, FTIR (Spectrum One spectrometer (series: 69288, Perkin Elmer, Schwerzenbach, Switzerland). All spectra were recorded from 4000–400 cm^−1^, with 64 scans.

#### 2.2.2. Contact Angle Measurements

The surface wettability of the PMMA_OA composites was tested by sessile drop method by contact angle device (EasyDrop Standard, Krüss, Hamburg, Germany, equipped with a monochrome interline CCD camera) using 20 μL of distilled water at room temperature 21 ± 1 °C. Water was dropped on the PMMA_OA composites’ surface using a microliter syringe (Hamilton Typ 1750 TLL). The results were given as an average value of a minimum 3 measurements and ±standard deviation (SD).

### 2.3. Antifungal Characterization of Composites

*Candida* Strain and Culture Conditions. 

This study used *C*. *albicans* ATCC90028 reference strain, obtained from the American Type Culture Collection (ATCC, Manassas, VA, USA); the stock was kept at –80 °C; the culture was maintained in Sabouraud 4% Glucose Agar (SGA; Sigma-Aldrich 84088, St. Louis, MO, USA).

#### 2.3.1. Biofilm Formation on PMMA_OA Composites 

*C*. *albicans* biofilm were formed on the PMMA_OA composites with modification of the previously described method [24,25,26,27,28]. 400 µL of the standardized inoculum 10^6^ cell/mL in RPMI medium was added over the composites placed in the 24-well tissue culture plates, previously sterilized under a UV C lamp for 15 min. Plates were covered with a lid, sealed with parafilm, and incubated at 37 °C for 24 h–period of biofilm formation. After incubation, 200 µL of *C*. *albicans* suspension in RPMI medium incubated with PMMA_OA composites (biofilm supernatant) were transferred to the new 24-well tissue culture plates. The rest of the suspension was discarded, and composites in 24 well plate were left to dry before XTT assay. 

#### 2.3.2. XTT Test on PMMA_OA Composites and in Biofilm Supernatant

XTT test was performed to quantify the metabolic activity of *C*. *albicans* biofilm on the composites as well as metabolic activity of planktonic *C*. *albicans* cells in medium incubated for 24 h with composites in two time intervals: at the same day and 6 days after composites’ preparation (T0 and T6, respectively). Briefly, XTT is prepared as a saturated solution at 0.5 g L^−1^ in sterile PBS (Dulbecco’s Phosphate Buffered Saline (DPBS • 1000)). Menadione was added to achieve a final menadione concentration of 1 μM in XTT solution before the experiment. Further, 200 µL of XTT/menadione was added on the PMMA_OA composite placed in the well of 24 well plates, as well as in 200 µL of medium transferred into new well plate. Plates were wrapped in aluminum foil to prevent light penetration and incubated at 37 °C for 3 h. After incubation, 100 µL XTT-menadione solution was transferred into new 96- well tissue culture plates. Changes in color intensity were measured with a micro plate reader (TECAN Infinite M200, Tecan, Männedorf, Switzerland) at 490 nm. 

### 2.4. Antifungal Susceptibility Test

#### 2.4.1. Determination of Minimal Inhibitory Concentration (MIC) 

The standard agar dilution method was modified to determine the lowest concentration of OA, which inhibits *C*. *albicans* growth [29]. Different concentrations of OA ranged from 0.0125, 0.025, 0.05, 0.1, 0.2, 0.4% were dispersed into 40 mL of Yeast Peptone Dextrose (YPD) agar in 50-mL Polypropylene flat tube. Per 1 mL of so-obtained agar containing a corresponding concentration of OA was added to a well of 24 well plate (4 wells per concentration). Control included agar without OA. After agar was solidified, 300 µL 10^6^ cell/mL *C. albicans* was added to its surface, and well plates were incubated at 37 °C for 24 h. After incubation, 100 µL of *C. albicans* suspension was transferred into new 96- well tissue culture plates and optical density was measured at 600 nm. 

#### 2.4.2. Embedded Filamentation Test (EFT)

In parallel with MIC, the effect of OA on the morphological appearance of *C. albicans* was tested in the embedded condition in agar. 50 µL 10^6^ cell/mL *C. albicans* were added in 5 mL of YPD agar containing the lowest and the highest concentration from the previous test (0.0125 wt% and 0.4 wt% of OA), in 50-mL Polystyrene Conical Tube (Sarstedt 352073). This suspension, mixed by inverting tube up and down, slowly and carefully to avoid making bubbles, was poured into the Petri dishes (Ø 30 mm). Plates were incubated at 37 °C for 24 h and 48 h. The morphological appearance of *C. albicans* species through the agar matrix was examined under an optical microscope (Nikon Eclipse Ti-E inverted microscope, Nikon Instruments Europe BV, Amsterdam, The Netherlands) after 24 h and 48 h.

#### 2.4.3. Statistic

Data obtained from the contact angle measurement, XTT assay, and cytotoxicity test were given as means ± SD. The significance of differences between more than two groups was analyzed with ANOVA followed by a post hoc Tukey’s test. Pearson’s correlation coefficient (r) was used to analyze associations between continuous variables. 

## 3. Results

### 3.1. Physico-Chemicals Characterization

#### 3.1.1. FTIR (Chemical Characterization of Composite Surface)

**Figure 1:**
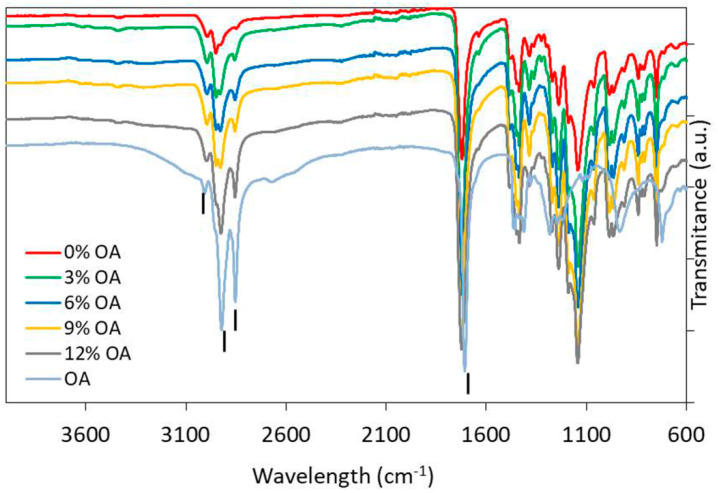
Fourier−transform infrared spectra of pure oleic acid, Poly(methyl methacrylate) and composites with 3%, 6%, 9%, and 12% (*w*/*w*) OA.

FTIR was performed on the composite-discs (PMMA_OA) with different OA concentrations as well as on the native materials (PMMA and OA) (Figure 1). In the spectrum of pure PMMA, bands at 2985 cm^−1^ and 2964 cm^−1^ can be attributed to the –C-H bond stretching vibrations of the –CH3 and –CH2–-groups, respectively [30]. The characteristic PMMA band corresponding to the stretching vibrations of the ester group appears at 1727 cm^−1^ (C=O) [31,32,33]. The bands observed in the PMMA spectra at 1437 cm^−1^ are assigned to the bending vibration of the C-H bond in (CH3) and the stretching of the ester group at 1147 cm^−1^ (C-O-C). The absorption band at 1243 cm^−1^ is due to the C-O-C stretching vibration. The bands at 1388 cm^−1^ and 754 cm^−1^ are due to vibration of the α methyl group. Characteristic absorption vibrations of PMMA can be observed at 1065 cm^−1^, 987 cm^−1^, 843 cm^−1^.

In the spectrum of native OA, the sharp bands at 2920 cm^−1^ and 2850 cm^−1^ can be assigned to C-H stretching in asymmetric and symmetric, respectively, of OA. Moreover, weak absorption bands at 3000 cm^−1^ may be attributed to =CH [34]. The bands that are related to the C=O and C–O stretch of COOH groups are found at 1710 cm^−1^ and 1285 cm^−1^, respectively [35,36,37].

The O-H in-plane and out-of-plane bands appeared at 1450 cm^−1^ and 930 cm^−1^, respectively. However, with the addition of OA in the PMMA, the peak at 930 cm^−1^ disappears.

Vibration peaks of –CH group of OA in region 2920 cm^−1^ and 2850 cm^−1^ overlaps with vibration of –CH group of PMMA spectra in the same region, but shifting of this group is noticed in the spectra of PMMA_OA composites and the peaks are more and more sharp with the increase of OA concentration between 2850 cm^−1^ and 2920 cm^−1^. Since all of the characteristic peaks of OA are visible in the composites, it can be concluded that OA is present in its native form on the surface of the composite. Also, the interactions between OA and PMMA are physical without new chemical bonding between functional groups of OA and PMMA.

#### 3.1.2. Water Contact Angle Measurement

**Figure 2:**
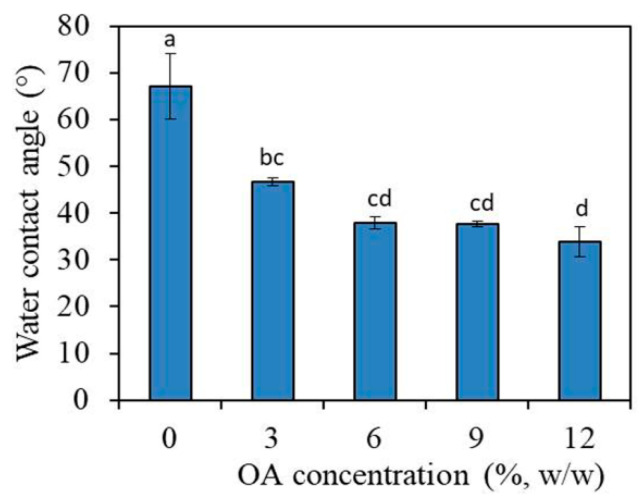
Water contact angle on the surface of composites with 0%, 3%, 6%, 9%, and 12% oleic acid. Bars indicate the mean values of water contact angles and vertical lines standard deviations (SD). Lowercase alphabetical letters above the columns show significant differences among the groups and compared to controls (*p* < 0.001, Tukey’s test). Results are presented as a mean ± SD.

Water contact angle on PMMA_OA composite-discs decreased from 67.6° for PMMA to 33.8° for PMMA_OA composites with 12% OA (Figure 2). Even for the composites with the lowest OA concentration, 3% OA in PMMA, the water contact angle was significantly decreased to 46.3° (*p* < 0.01), compared to 67.6° (0% OA in PMMA).

The results showed that water contact angle decreased with the increase of OA concentration, indicating that OA changes the surface properties of PMMA in hydrophilic PMMA_OA composites.

### 3.2. Antifungal Characterization

#### 3.2.1. Antifungal Characterization of PMMA_OA Composites with XTT Test

**Figure 3:**
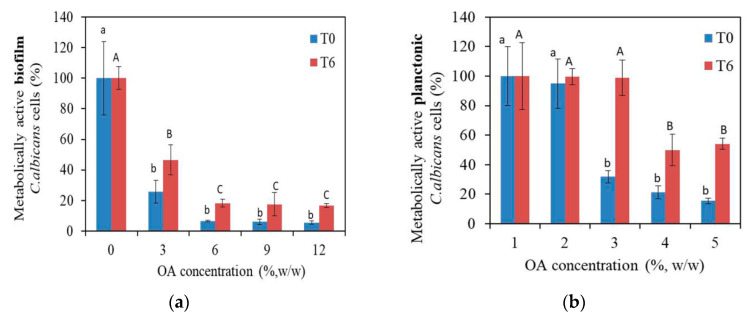
Metabolic activity of *C. albicans* cells on the surface of composites with 0, 3, 6, 9, and 12 wt% of oleic acid (OA) in Poly(Methyl Methacrylate), (**a**) in biofilm and (**b**) in the medium above composites in 2-time points: T0 and T6. Bars indicate the mean values and vertical lines standard deviations (SD). Different lowercase alphabetical letters above the columns show significant differences among the groups at T0 time (*p* < 0.001, Tukey’s test). Different uppercase alphabetical letters above the columns show significant differences among the groups at T6 time (*p* < 0.001, Tukey’s test). The results were presented as mean ± SD compared to control.

The metabolic activity of both planktonic and biofilm *C. albicans* cells incubated with PMMA_OA composites compared to the metabolic activity of planktonic and biofilm cells in the control group (incubated with PMMA without OA) is given in Figure 3. In PMMA_OA composites, a biofilm formation was statistically and significantly decreased even at the lowest OA concentration, (3% OA in PMMA), compared to metabolically active biofilm cells on PMMA. Typically, the percentage of metabolically active biofilm cells was 25.80% on composites with 3% OA. The test was performed in the two different time points (T0 and T6) to study the antifungal surface properties of PMMA_OA composites with time. Moreover, on the composites with 3% OA, antifungal activity was lower (46.58% metabolically active biofilm *C. albicans* cells) in the T6 time point than in the T0 time interval (25.80%). Biofilm formation in T6 decreased on all PMMA_OA composites with ≥6% OA having less than 15% of the metabolically active *C. albicans* cells compared to that on PMMA in T6. In the T0 time point, at the highest OA concentrations of 9% and 12% OA in PMMA, the percentage of metabolically active planktonic *C. albicans* cells was 21.36% and 15.46%, respectively. In the T6 time point, metabolic activity was higher compared to T0, with ~50% metabolically active planktonic *C. albicans* cells.

#### 3.2.2. Antifungal Susceptibility Test

In order to test the effect of OA on *C. albicans* growth and morphology, the following two tests were employed:

##### Determination of Minimal Inhibitory Concentration (MIC) 

**Figure 4:**
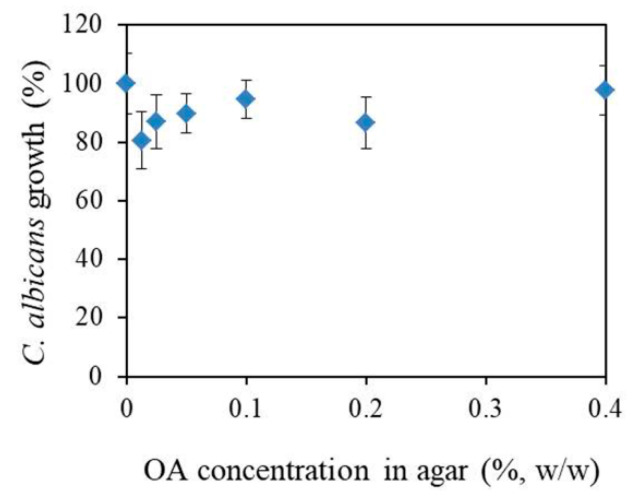
Oleic acid (OA) affection on the *C. albicans* growth 24 h of incubation in agar loaded with different oleic acid (OA) concentrations. Results are given as mean ± standard deviation percentages of A_620_ readings compared to control (0% OA) as a function of OA concentration.

The susceptibilities of planktonic *C. albicans* cells to OA were examined by measuring of optical density of *C. albicans* suspension on the agar surface containing different OA concentrations. The results show that OA did not inhibit *C. albicans* growth (Figure 4).

##### Embedded Filamentation Test (EFT)

**Figure 5:**
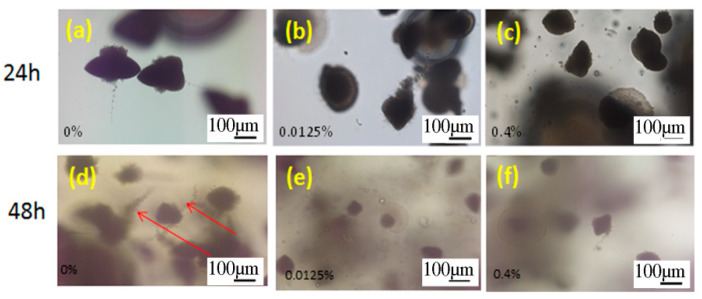
Optical micrographs of the *C. albicans* cells after 24 h (**a**,**b**,**c**) and 48 h (**d**,**e**,**f**) of incubation, embedded in agar without oleic acid (OA), (control, 0% OA, (**a**,**d**)), with 0.0125% OA (**b**,**e**) and 0.4% OA (**c**,**f**). Arrows mark spindle-shaped colonies with sporadic hyphae and/or pseudohyphae on the margins and lateral yeasts after 48 h incubation.

This test was carried out to study if OA affects a *C. albicans* cells morphology when embedded in YPD agar and incubated for 24 h and 48 h at 37 °C. The OA concentrations in agar were chosen according to the previous test (i.e., the highest concentration in this test corresponds to the highest concentration in the previous test, 0.4% OA). The morphology of *C. albicans* was assessed under an optical microscope. In embedded condition in YPD agar without OA, after 24 h incubation, *C. albicans* cells formed spindle-shaped colonies, including mainly the yeast cells, while rare hyphae and/or pseudohyphae could be observed peripheral on the colony, as it has been reported previously [23,38,39]. After 48 h of incubation, *C. albicans* cells formed the spindle-shaped yeast colonies with the formation of numerous hyphal branches and lateral yeasts derived from the colonies, which was in agreement with the previous study [23]. However, in agar with both tested OA concentrations, there was no hyphal formation neither after 24 h nor 48 h of incubation (Figure 5).

## 4. Discussion

The widespread application of PMMA in dentistry has driven biomaterial research to overcome challenges related to biofilm formation on medical devices made from PMMA, such as dentures [40]. PMMA dentures are that it is a suitable substrate for *C. albicans* adhesion and biofilm formation on dentures due to its hydrophobic nature and rough surface texture [19]. Moreover, it is challenging to treat *C. albicans* when it forms a biofilm due to its increasing resistance to conventional antifungal drugs.

Different approaches have been proposed to prevent *C. albicans* adhesion and biofilm formation on dentures by making them antifungal. This can be achieved by incorporating an antifungal compound such as chlorhexidine, fluconazole, amphotericin B, or nystatin into denture PMMA [13,14,17,18,20]. However, increased tolerance and resistance of *Candida* spp. to used antifungal drugs compromise successful treatment [7,25,41,42]. Thus, the advantages of using plant-derived compounds as therapeutic agents include fewer adverse effects, lower chances of antimicrobial resistance, and better efficiency in controlling biofilm-related infections [21]. 

In this context, the present study examined the inhibitory potential of OA incorporated into PMMA matrix on *C. albicans* biofilm formation on the surface of composites. Results showed that antibiofilm surface properties of PMMA_OA were due to the presence of OA on its surface (confirmed with FTIR analysis) and its inhibitory effect on *C. albicans* filamentation, a key step in biofilm formation. 

The goal of this study were to analyze how OA affects surface properties of PMMA and how PMMA_OA composites surface will affect further *C.albicans* attachment and biofilm formation. OA is an unsaturated fatty acid insoluble in water. OA has a hydrophilic, polar head and a hydrophobic tail. OA’s polar carboxyl (COOH) group is soluble, while the tail is insoluble in water. The possible reason for changing of PMMA_OA surface properties in hydrophilic one could be the orientation of OA molecule in the matrix, wherein the polar head would be orientated on the surface while the tail would be orientated out of the surface [23,43]. However, according to Garland et al. vapor-deposited OA on both polar (silica) and nonpolar (polystyrene) substrates resulted in the hydrophobic surface at high coverages of OA which suggests that the hydrocarbon chain on the OA molecule is facing away from the surface [43]. Hence, the challenge was to test if hydrophilic surface of PMMA_OA composites affects further *C. albicans* attachment and biofilm formation.

It has been reported that hydrophilic surfaces reduce fungal adhesion and the consequent biofilm formation to polymeric biomaterials [19,40,44]. In this regard, the correlation between the water contact angle of the PMMA_OA and the percentage of metabolically active biofilm cells attached to the composite surface was determined [44]. We showed a positive correlation (r = 0.987) between the value of water contact angle and the percentage of metabolically active biofilm cells on PMMA_OA composites. It means that the addition of OA into PMMA affects the changes of wetting properties of the surface (in hydrophilic one) and significantly decreases the percentage of metabolically active biofilm cells in all samples of PMMA modified with all tested OA concentrations. Therefore, the decreased biofilm formation on the PMMA_OA composite surface could be a consequence of the combined action of the antibiofilm surface properties of the PMMA_OA and the increase in surface hydrophilicity. 

Furthermore, we showed that PMMA_OA composites decrease metabolic activity of planktonic cells, suggesting that OA may be released from composites and influences planktonic cells as well.

In contrast to our study that incorporation of OA into PMMA affects metabolic activity of both biofilm and planktonic cells, Muthamil et al. have recently reported the non-fungicidal effect of OA against *Candida* spp. by XTT assay. According to their study, incubation of *Candida* in spider broth in the presence of OA (at different concentrations 5, 10, 20, 40, 80, 160 and 320 μg mL^−1^), resulted in a thinner biofilm formed on glass slides with reduced biomass and architecture of mature biofilm compared to the controls (in the absence of OA) [21]. 

Therefore, to understand the mode of action of OA when it is not being incorporated into PMMA, we studied the effect of OA on the growth and morphology of *C. albicans* cells in agar. In our study, OA was not dissolved in any non-aqueous solvent to avoid the possible influence of non-aqueous solvents on *C. albicans*, but it has been dispersed in agar. Thus, we studied the effect of, pure” OA on *C. albicans* cells after incubation on an agar surface containing different OA concentrations. Results showed that OA did not inhibit *C. albicans* growth, which was in agreement with Muthamil et al. (2020) [21]. However, Lee et al. (2020) have reported that OA inhibits the growth of *C. albicans*, but at higher concentration, such as >500 μg mL^−1^ [22].

In this study, the filamentation test showed that even the highest OA concentration, previously tested for MIC (0.4%), did not inhibit *C. albicans* growth, but it prevented hyphae formation (filamentation) after 24 h and 48 h of incubation. Similarly to reports of proved antibiofilm effects of some fatty acid at concentrations lower of their MICs, suppression of *C. albicans* biofilm formation occurs by inhibiting hyphal growth and cell aggregation [22]. Additionally, it has been shown that OA treatment could significantly reduce the extracellular polymeric (EPS) matrix’s carbohydrates, lipids, and eDNA content of the EPS matrix [21], which protects biofilm cells from the host immune system and the antifungal agents. Given that OA changes the ergosterol content of *Candida* spp., it qualifies it as a more potent drug than standard antifungal agents [21].

Within the limitations of this in vitro study, the first results of here developed PMMA_OA composites focusing on its physico-chemicals characterization and antifungal properties have been reported. However, the morphological analyses of the samples with SEM should be performed in future research to provide more information how OA incorporation into PMMA affect surface properties of PMMA_OA composites. Furthermore, OA is non-toxic compound [45,46] and a potential of PMMA_OA composites for biomedical applications could be considered.

## 5. Conclusions

This study demonstrates, for the first time, incorporation of OA into PMMA and development of PMMA_OA composites with antibiofilm surface properties. This new PMMA_OA composite with ≥3% OA significantly reduces metabolic activity of biofilm cells even six days after PMMA_OA preparation. Moreover, OA present on the composites surface, results in increased hydrophilic surface properties of this developed composites. This study confirmed that OA prevents filamentation and, consequently, the early stage of *C. albicans* biofilm formation on PMMA_OA composites surface. Since OA is naturally occurring non-toxic molecule and has no antimicrobial resistance, it could be a promising agent for modifying dental material such as denture and preventing the *Candida* associated denture stomatitits. Within the limitations of this study, it can be concluded that PMMA_OA may be used as a dental polymer to reline inner surface of denture having the potential to prevent and to treat *Candida* associated infection in denture wearers. For this purpose. further research is required to evaluate additional biological and mechanical parameters of PMMA_OA for clinical applications.
